# Explainable stress type classification captures physiologically relevant responses in the Maastricht Acute Stress Test

**DOI:** 10.3389/fnrgo.2023.1294286

**Published:** 2023-12-05

**Authors:** Jaakko Tervonen, Johanna Närväinen, Jani Mäntyjärvi, Kati Pettersson

**Affiliations:** VTT Technical Research Centre of Finland Ltd., Espoo, Finland

**Keywords:** interpretable artificial intelligence, machine learning, physiology, stress, acute stress detection

## Abstract

**Introduction:**

Current stress detection methods concentrate on identification of stress and non-stress states despite the existence of various stress types. The present study performs a more specific, explainable stress classification, which could provide valuable information on the physiological stress reactions.

**Methods:**

Physiological responses were measured in the Maastricht Acute Stress Test (MAST), comprising alternating trials of cold pressor (inducing physiological stress and pain) and mental arithmetics (eliciting cognitive and social-evaluative stress). The responses in these subtasks were compared to each other and to the baseline through mixed model analysis. Subsequently, stress type detection was conducted with a comprehensive analysis of several machine learning components affecting classification. Finally, explainable artificial intelligence (XAI) methods were applied to analyze the influence of physiological features on model behavior.

**Results:**

Most of the investigated physiological reactions were specific to the stressors, and the subtasks could be distinguished from baseline with up to 86.5% balanced accuracy. The choice of the physiological signals to measure (up to 25%-point difference in balanced accuracy) and the selection of features (up to 7%-point difference) were the two key components in classification. Reflection of the XAI analysis to mixed model results and human physiology revealed that the stress detection model concentrated on physiological features relevant for the two stressors.

**Discussion:**

The findings confirm that multimodal machine learning classification can detect different types of stress reactions from baseline while focusing on physiologically sensible changes. Since the measured signals and feature selection affected classification performance the most, data analytic choices left limited input information uncompensated.

## 1 Introduction

Stress affects people both positively and negatively: in some cases it helps individuals cope while in others it paralyzes them. The human body adapts to stressful situations efficiently but when the transient stress repeats without adequate recovery, it can convert slowly and insidiously into chronic stress that threatens health and wellbeing. Therefore, studying stress detection methodology has received significant attention in recent years. Current methods, however, are limited since they focus on binary detection between stress and non-stress states (Vos et al., 2023). In addition, the various machine learning (ML) components affecting detection performance remain understudied in conjunction with a physiological inspection of the most important features to apply.

The harmful effects of stress are mainly associated with frequent activation of the hypothalamus-pituitary-adrenal (HPA), which triggers a sequence of events causing the release of cortisol in the bloodstream, increasing cortisol levels (Sharpley, 2009). The stressful activation may also begin from the sympathetic nervous system through sympatho-adrenaline-medullary (SAM), which causes the increase of arousal level via adrenaline (Sharpley, 2009; Smeets et al., 2012). Thus, two stress-responsive axes evoke physiological changes. These can be detected from biosignal features such as heart rate (HR) and heart rate variability (HRV) from electrocardiogram (ECG), skin conductance responses from electrodermal activity (EDA), spectral power parameters from electroencephalogram (EEG), and eye movement and blink statistics from electro-oculogram (EOG) (Schmidt et al., 2019; Giannakakis et al., 2022; Vanhollebeke et al., 2022).

A vast majority of stress detection research is conducted in laboratory conditions, where stress must be induced. The induction protocols may contain social-evaluative, mentally and attentionally engaging, or physically uncomfortable elements (Dickerson and Kemeny, 2004; Schmidt et al., 2019). Several studies, such as Mozos et al. (2017), Schmidt et al. (2018), and Aristizabal et al. (2021), have employed the Trier Social Stress Test (TSST) (Kirschbaum et al., 1993), which comprises a public speaking and a mental arithmetic (MA) task. A cold pressor test (CPT), where participants hold their right hand in cold water for a short period (1–3 min), has been used to deliver physical stress (Daniels and Georgiou, 2019; Ghiasi et al., 2020; Mishra et al., 2020a). These protocols have typically had resting sessions between the different stressors and each subtask has been completed just once. However, such setups are far from real life situations where the various stressors offer no chance to rest and recover between them.

In contrast, the Maastricht Acute Stress Test (MAST) (Smeets et al., 2012) consists of alternating trials of CPT and MA subtasks, each lasting 45–90 s, repeated for 10 min with no resting sessions between the trials. By design, MAST contains two stressors (cognitive MA and physical CPT), but it also contains elements of a social-evaluative stressor since the researcher pressures the participant to perform better and wrong answers cause a penalty in the arithmetic subtask. The different stressors cause different physiological reactions in the human body, which are briefly reviewed next.

Stressors including social-evaluative or cognitive elements increase blood pressure, HR, blink rate, and EDA activity, decrease HRV (Schuri and von Cramon, 1981; Hellhammer and Schubert, 2012; Aristizabal et al., 2021), and cause significant changes in some spectral EEG parameters (Vanhollebeke et al., 2022). Since CPT triggers a sympathetic activation (Mourot et al., 2008), it causes changes in EDA (Mourot et al., 2008; Ghiasi et al., 2020) and in blink rate (Paparella et al., 2020). However, the HR response to the CPT varies individually: Mourot et al. (2008) reported that HR increased continuously during the 3-min CPT for 20 out of 39 participants and decreased for the others after a short initial increase. As for EEG, most studies with CPT have been related to pain research and Chang et al. (2002) summarize these findings: decreased alpha band and increased beta band activity. They also observed increased frontal theta activity and decreased posterior alpha activity during a three-minute CPT. Recently, Chouchou et al. (2021) concluded that the decrease in parietal alpha activity is a robust but unspecific marker of pain.

In earlier studies, the MAST has been mainly treated as a single 10-min stressor that increases blood pressure and salivary cortisol levels (Smeets et al., 2012; Quaedflieg et al., 2017; Shilton et al., 2017). However, HR evidenced no group level effect between pre-MAST and post-MAST phases (Shilton et al., 2017). The limited reactivity could mean that the HR reactions have subsided prior to post-MAST measurements, as reported in a TSST study (Hellhammer and Schubert, 2012). As MAST consists of a dynamic sequence of two stressor trials, it would be meaningful to treat the stressors as two distinct states rather than as a single block of general stress. Verifying whether these two stressors elicit different types of stress either through HPA or SAM activation would be complicated due to the need for hormonal measurements between the repeated trials and the leakage arising from the missing recovery periods. Nevertheless, the current study refers to the induced states as stress types since the two stressors are expected to cause varied physiological reactions in the human body. This leads to stress detection problem with two different stressors, physical stress (CPT) and cognitive stress with elements of social stress (MA). The problem is challenging, primarily because there is no recovery between alternating MA and CPT subtasks, and there is likely to be physiological leakage, especially affecting the classification of the first few subtask windows. So far, few studies have used such an approach to study the stress and physiology during the MAST but it has been shown in Pettersson et al. (2020) and Tervonen et al. (2021b) that CPT and MA can be separated with ML methods from biosignal measurements.

A ML approach to stress detection follows a processing pipeline with steps such as data preprocessing, feature extraction, and classification (Schmidt et al., 2019). Preprocessing consists of synchronization of the available data streams, filtering the raw signals to increase the signal-to-noise ratio, and segmenting the signals into desired lengths to enable classification of measurements. At feature extraction, the filtered and segmented signals are turned into more insightful features, e.g., HR is computed from the ECG signal. Finally, the features are classified with a ML algorithm combined with proper validation procedure to ensure generalizability of the model.

Despite the different types of stressors and resulting stress, existing studies focus on binary stress detection (Vos et al., 2023). Even when the stress induction protocol has included a variety of social-evaluative or cognitive stress stimuli, the divergent stressful conditions have been labeled as a single stress state, e.g., Mishra et al. (2020b) and Chalabianloo et al. (2022). The works with a non-binary setup have detected the intensity of stress (Gjoreski et al., 2017), classified between resting, mental stress, and physical activity (Chalabianloo et al., 2022), or between resting, stress, and an affective state (Feng et al., 2023) instead of detecting the type of stress.

Additionally, despite the comprehensive and versatile reactions that stress causes in the human body, the emphasis in biosignal based stress detection studies is in HRV and EDA measurements (Schmidt et al., 2019; Giannakakis et al., 2022; Vos et al., 2023). The studies utilizing EEG often use spectral analysis of multichannel EEG measurements (Vanhollebeke et al., 2022), which is time-consuming to set up, computationally heavy and infeasible in real-life situations. A more realistic option is to measure brainbeat, a derived EEG parameter introduced by Holm et al. (2009). It is computed from the frontal and parietal signals, which can be measured with simple wearable EEG devices. EOG is commonly measured in multichannel EEG studies to remove eye movement and blink artifacts from the EEG signal. However, the EOG signals have rarely been used as a parameter in stress detection, despite that eye movements and blinks react to changes in human cognitive state (Marshall, 2007; Henderson et al., 2013; Pettersson et al., 2020).

Consequently, the knowledge of which physiological features to apply in stress detection is incomplete. Despite that feature importance analysis is essential to properly interpret and validate a classification model, the exact features that were the most important are scarcely reported. This objective was progressed in Pettersson et al. (2020) and Tervonen et al. (2021a,b), but they employed large sets of features and feature importance was computed with methods that may produce model-specific or biased estimates (e.g., partial dependence plots in Tervonen et al., 2021a which is based on feature permutations, disregarding feature intercorrelations). Shapley additive explanations (Lundberg and Lee, 2017) would be a more robust choice, as they fairly assign the prediction to individual features. The approach was adopted in stress detection context in Chalabianloo et al. (2022) but the analysis was limited to just HRV and EDA features. The existing investigations of feature importance have also been rather technical (Tervonen et al., 2021a,b; Chalabianloo et al., 2022) and physiological sensibility is little discussed.

On the modeling side, the focus on stress detection has been on improving the data processing pipeline and especially on conducting extensive evaluations to find the best classifier and signals to measure (Schmidt et al., 2019; Giannakakis et al., 2022; Vos et al., 2023). Thus, only the results with several classifiers and signal combinations are often reported, e.g., Schmidt et al. (2018) and Pettersson et al. (2020), and little attention is given to estimating the effect of other modeling choices, such as data segmentation and optimizing the classifier hyperparameters. Therefore, assessing the importance of conducting those steps is hindered.

The present study investigates the physiological responses in ECG, EDA, EOG, and EEG to the subtasks of the MAST protocol. The main aim is to (1) determine whether reactions to the stressors deviated with a mixed model analysis, and (2) detect the stress types from a baseline condition using a ML analysis. The emphasis on the ML analysis is on evaluating the necessity and effect of different components in the pipeline, namely which signals to measure and classifier to use, the duration of feature windows, feature selection, and hyperparameter optimization. Finally, the study examines physiological parameters' impact on classification, both with an extensive set of features and an interpretable one of a few easily interpretable features, and reflects these results to physiology. To the best of authors knowledge, this study is the first to extensively examine the physiological responses during the MAST with both a statistical and an explainable ML analysis.

## 2 Materials and methods

The analysis in this study was conducted as follows: (1) the data were collected; (2) the data were preprocessed and features extracted; (3) the data were analyzed with mixed models to determine whether the protocol elicited varying reactions to the MAST subtasks and to see how the physiological features reflected stress; and (4) a ML analysis was conducted to find whether the stress types can be classified and model behavior was explained to see which exact features affected classification the most and how.

### 2.1 Data collection

Study participants were healthy volunteers (*N* = 24, 7 male) with a mean age of 23.5 years (sd = 3.0 years, range 19–29). The volunteers were required to be right-handed, have no history in cardiac disorders, no severe depression, and no consumption of medicines affecting the autonomous nervous system. The participants were instructed to maintain their normal lifestyle, avoid any atypical activities during the 24 h before the study visit and ensure they do not arrive hungry nor very full. All visits were hosted by the same researcher and the experiments were conducted one participant at a time. All participants gave their informed consent in all the studies. The participants were also informed that they could stop their participation at any given time and their participation was completely voluntary. The study proposal was evaluated by the Ethics Committee in the Humanities and Social and Behavioural Sciences of the University of Helsinki.

**Figure 1:**
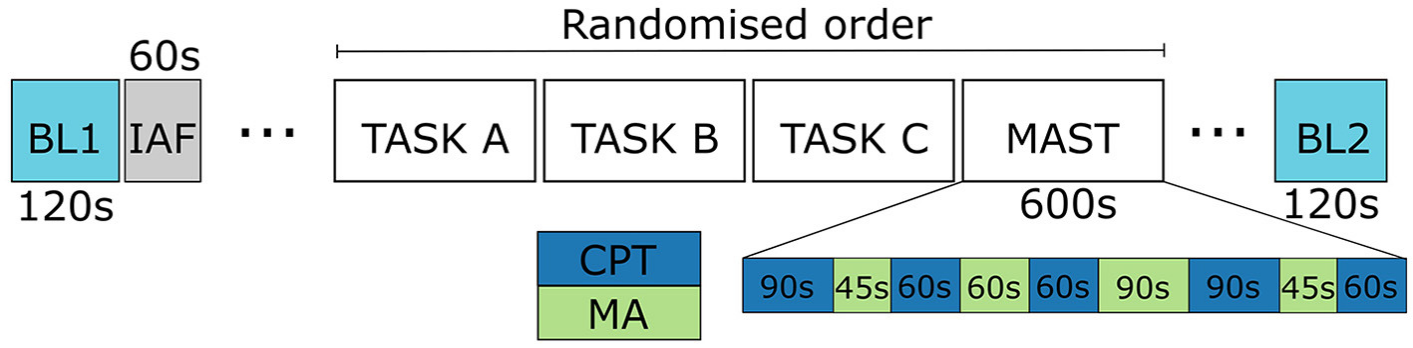
The study protocol. Only the data from baselines (BL1 and BL2), and MAST are discussed in this work. IAF stands for the phase when individual alpha frequencies were measured.

The MAST was conducted as part of a larger protocol (see Figure 1). Baseline physiology was recorded for 120*s* before and after the protocol while the participants stayed still and kept their eyes on a fixation point on a computer screen. These baseline periods served as the baseline resting state in this study.

The MAST protocol consisted of 10 min of alternating trials of physical (CPT) and psycho-social (MA) stress, each trial with a varying duration between 45 and 90 s. In the cold immersion, the participants held their hand in water with a constant temperature of 2°*C*. The participants counted down from 2,043 in steps of 17 under time pressure and penalization in the arithmetic subtask MA; the researcher urged them to be fast and they had to return to start when making a mistake. The instructions for each subtask was visible on a computer screen during the whole protocol.

Physiological signals were collected with NeurOne system (Bittium, Oulu, Finland). The measured signals included one-channel ECG between left collarbone and right lower back, EDA between the index and middle finger, 64-channel EEG placed according to the International 10-20 system, and two-channel EOG between the electrodes placed above and beneath the left eye (vertical) and the outer corners of the eyes (horizontal). Electrodes T9 and T10 served as a linked mastoid reference. The participants held their left hand still during the experiment to ensure undisturbed EDA signal. Each signal was sampled at a sampling rate of 1, 000*Hz* and low-pass filtered with a cut-off frequency of 250*Hz*.

### 2.2 Data preprocessing and feature extraction

Data processing followed a feature-based approach, where the raw biosignals were processed into features usable for classification of stressful states. A total of 150 features were computed, and Table 1 lists all the extracted features and provides the names of features used later in text.

**Table 1:** List of extracted features from each signal, and their naming convention used in other figures and tables. Features written in bold denote interpretable features.

**Feature name prefix**	**Features**	**Description**
ecg_hr_	**mean**, std, lq, uq, median, min, max, range, cv	Statistics of HR
ecg_hr_d1_	mean, std, lq, uq, median, min, max, range, cv	Statistics of the 1st derivative of HR
ecg_hrv_	**ibi_mean**, ibi_median, ibi_range	Statistics of IBIs
ecg_hrv_	sdnn, sdsd	Std of IBIs and successive differences
ecg_hrv_	(p)nn20, (p)nn50	Percentage and number of IBIs differing more than 20ms/50ms
ecg_hrv_	**rmssd**	Root mean square of successive differences
ecg_hrv_	cvnni, cvsd	Ratio of sdnn and mean IBI, and RMSSD and mean IBI
ecg_hrv_	vlf, lf, hf, total_power	Power in very, low, high frequency bands and total power
ecg_hrv_	lf/hf	Ratio of lf and hf
ecg_hrv_	lfnu, hfnu	Normalized lf and hf
ecg_hrv_	hrvti	HRV triangular index
ecg_hrv_	cvi, csi, Modified_csi	(Modified) cardiac sympathetic index, cardiac vagal index
ecg_hrv_	sd1, sd2, sd2/sd1	Poincar plot std perpendicular and along the identity line, their ratio
eda_phasic_	**mean**, std, lq, uq, median, min, max	Statistics of phasic EDA
eda_phasic_d1_	mean, std, lq, uq, median, min, max	Stats of the 1st drv. of phasic EDA
eda_phasic_d2_	mean, std, lq, uq, median, min, max	Stats of the 2nd drv. of phasic EDA
eda_phasic_	**npeaks**	Number of peaks
eda_phasic_	risetime, dectime	Time signal increased/decreased
eog_blink_br_	**mean**, std, lq, uq, min, max, median, rmssd, cv, kurtosis, skewness, p5, p95	Statistics of blink rate
eog_blink_bdur_	**mean**, std, lq, uq, median, rmssd, cv, kurtosis, skewness, p5, p95	Statistics of blink duration
eog_blink_tbb_	**mean**, std, lq, uq, median, rmssd, cv, kurtosis, skewness, p5, p95	Statistics of time between blinks
eog_blink_bskew_	**mean**, std, lq, uq, median, rmssd, cv, kurtosis, skewness, p5, p95	Statistics of blink waveform skewness
eog_sac_sr_	**mean**, std, lq, uq, min, max, median, rmssd, cv, kurtosis, skewness, p5, p95	Statistics of saccade rate
eog_sac_sdur_	**mean**, std, lq, uq, median, rmssd, cv, kurtosis, skewness, p5, p95	Statistics of saccade duration
eog_sac_tbs_	**mean**, std, lq, uq, median, rmssd, cv, kurtosis, skewness, p5, p95	Statistics of time between saccades
eeg_	**bb**	Brainbeat

Std, standard deviation; lq and uq, lower and upper quartile; pX, the X'th percentile; sac, saccades.

Heartbeats and interbeat intervals (IBIs) were extracted from the ECG signals with a Matlab toolbox (Sedghamiz, 2014) implementing the Pan-Tompkins algorithm. Then, HR and HRV features were computed from the IBIs. The statistics of HR and its first derivative were calculated. HRV was extracted in time, frequency and non-linear domain with the Python package *hrv-analysis* (Champseix, 2018). In total, 44 features from the ECG signal were extracted.

Saccades and blinks were extracted from the EOG signals with an automated algorithm (Pettersson et al., 2013). Once obtained, the statistics of saccade rate, time between saccades, saccade duration, blink rate, time between blinks, blink duration, and blink waveform skewness were computed for a total of 81 features.

The EDA signal was decomposed into phasic and tonic component using the Ledalab Matlab software (Benedek and Kaernbach, 2010). Then, statistics of the phasic component and its two first derivatives were computed, along with the number of skin conductance responses (SCR) and the total time the phasic component was increasing and decreasing, totalling 24 features.

Finally, the brainbeat (BB) was extracted from the EEG signal as follows. The EEG data were bandbass (1 − 40*Hz*) filtered. The eye movement artifacts were removed by regression algorithm, the EEG signal was down-sampled to 200 Hz and Fourier transformed to yield spectra. First, individual alpha frequencies (IAFs) were extracted from eyes closed condition (measured right after the baseline in the beginning of the measurement protocol) for each participant and the individual alpha band range was set to IAF 2*Hz* and theta range 48*Hz*. The BB parameter was computed as the ratio theta(Fz)/alpha(Pz) (Holm et al., 2009).

To analyze the effect of the selected window duration, all features were extracted in windows of 10, 15, 30, and 45 *s* with a window slide of 5, 7.5, 15, and15*s*, respectively. The window durations and slides were selected to fit the length of the MAST subtasks to minimize data loss. Shorter windows than 10*s* were not considered, since many of the features were computed from detected events: e.g., in Bentivoglio et al. (1997) it was observed that mean blink rate in rest condition is 17 blinks per minute, which is approximately one blink every 4*s*. Thus, shorter windows might not contain any blinks. Moreover, no saccades were detected in some, especially shorter, windows. The saccades made may have been too small to detect them from the EOG signal, lost in transient noise, or nonexistent, as the participants were instructed to look at a fixation point in the BL task so they should not have made saccades. Windows with too few blinks or saccades were left out of the analysis. Table 2 lists the number of samples at each window length; see Section 2.4.1 for a description of feature sets.

**Table 2:** Number of samples in each feature set at each window length. Missing values occurred mainly when participants made too few blinks or saccades to compute EOG features.

**Feature set**	**Window length (** ***s*** **)**			
	**10**	**15**	**30**	**45**
ECG	3,762	2,420	1,078	814
EDA	3,762	2,420	1,078	814
EOG	2,520	1,956	1,012	792
BB	3,762	2,419	1,077	813
ECGEDA	3,762	2,420	1,078	814
EOGBB	2,519	1,954	1,010	790
ECGEDAEOGBB	2,519	1,954	1,010	790

All the features extracted may be important for classification, but some of the features, such as statistics of second derivatives, are difficult to interpret. Therefore, an interpretable set of features was defined, consisting mainly of average measures: the mean of HR, IBI, phasic EDA, blink rate, time between blinks, blink duration, blink skewness, saccade rate, time between saccades, and saccade duration. In addition, root mean square of successive differences (RMSSD, a HRV measure), number of SCRs and BB were added. This set was used for an explainable machine learning analysis and a mixed model analysis.

To account for the subjective nature of physiological measurements, the features were normalized by person specific *z*-score standardization which has been shown to outperform other normalization strategies (Gjoreski et al., 2020).

### 2.3 Mixed model analysis

The physiological stress reactions during the MAST were explored with a linear mixed model analysis. The models were applied to assess whether the protocol elicited different kinds of stress (rest = BL, physiological stress = CPT, psycho-social stress = MA) using features from the biosignals that have been found to reflect stress or cognitive loading: ECG (heart rate, inter-beat-interval; ibi, rmssd), EDA (number of peaks, mean), EOG (blink rate, time between blinks, blink duration, blink skewness, saccade rate, time between saccades, saccade duration) and EEG (brainbeat).

The subtask type represented a fixed effect, participant a random effect, and a 45 *s* window with a 15*s* window slide was selected for all features. The models were computed with *R*'s lmer and glmer functions from the lme4 package (Bates et al., 2015). Since the number of EDA peaks reflects counts, a generalized linear mixed model (glmer) with Poisson link and maximum likelihood with Laplace approximation was applied in the fit for it and *p*-values were computed using asymptotic Wald test. As for the other features, a linear mixed model (lmer) with maximum likelihood estimation was employed and *p*-values were computed with Satterthwaite's method (Luke, 2017). The null models were compared to stress type models with analysis of variance (ANOVA) and the *post-hoc* analyses were executed by using Tukey's contrasts. The significance level for statistical inference was Bonferroni corrected to account for multiple testing.

### 2.4 Machine learning analysis

#### 2.4.1 Classification

Seven classifiers commonly used in stress detection studies detected CPT and MA from BL: k-nearest neighbors (KNN), linear and quadratic discriminant analysis (LDA, QDA), support vector machine (SVM), decision tree (DT), random forest (RF), and extreme gradient boosting (XGB), each of which is shortly described in the next paragraph.

KNN serves as an example of a distance-based classifier. A new observation is classified to the class, which most *K* closest training observations come from. LDA, QDA, and SVM construct hyperplanes to separate the classes. QDA assumes that the features follow a multivariate Gaussian distribution within each class and classifies a new observation to the class with the highest posterior probability, which gives rise to a quadratic decision boundary. LDA is similar to QDA, but it further assumes that covariance matrices of the different classes are equal, giving rise to a linear decision boundary. On the other hand, SVM makes no assumptions on the probability distributions but instead it directly constructs an optimal hyperplane with maximal margin to the different classes, combined with transformation to a space in which the features are linearly separable. DT, RF, and XGB are tree-based classifiers, which split the data hierarchically with an aim to contain observations from a single class only in each leaf node. RF consists of several independent DTs, each of which is trained on a random subset of samples and features, whereas in XGB, the DTs are not independent but try to fix the errors made by previous trees. For further reading on the classifiers, we refer to Hastie et al. (2009).

Classification was performed with seven feature sets to assess the effects of signal types. The feature sets were formed through feature-level fusion, i.e., features from individual signals were extracted independently and fused prior to classification. First, each signal distinguished between the stress states alone (feature sets ECG, EDA, EOG, BB). Next, ECG and EDA features were combined (feature set ECGEDA) since many devices (e.g., Empatica E4 and Shimmer3) enable measuring those together. Similarly, EOG and BB were fused (feature set EOGBB) since EOG is often measured with EEG, to remove eye movement artifacts from the EEG signal. Finally, all available signals were used together (feature set ECGEDAEOGBB). Table 2 displays the number of samples for each feature set and window length.

Classification performance is generally improved by hyperparameter optimization and feature selection. In this work, Bayesian optimization was applied to find optimal hyperparameters for each classifier (Bergstra et al., 2013). Non-informative priors with reasonable value range were adopted for each parameter, and the optimization was continued for 50 iterations per classifier, expect for LDA and QDA, which have no optimizable parameters. In addition, features were selected using the sequential forward floating search (SFFS) algorithm (Pudil et al., 1994), which adds or removes features as needed one by one until convergence. Feature selection was not conducted for tree-based models because they can simply ignore non-informative features during model training nor for the BB feature set, which consisted of a single feature.

To estimate how well the models generalize to new users, a leave-three-out validation strategy was employed. At each iteration, the data of three users was left out, the model was trained with the rest of the users' data and evaluated with the left-out fold. Additionally, a nested leave-three-out validation loop was included for hyperparameter optimization and feature selection to ensure that optimal parameters and features were selected according to validation, and not testing data. Thus, at each validation iteration, an internal leave-three-out validation was conducted to select the features and the best hyperparameters. Balanced accuracy, weighted by number of samples in each fold, served as an optimality criterion, since the label distribution was slightly skewed: as computed from subtask durations (see Figure 1), the data consisted of (up to a rounding error) 28.6% BL, 42.9% CPT, and 28.6% MA. The results are reported in terms of average balanced accuracy and its standard deviation across the validation folds.

In terms of computation time, one validation iteration lasted on average for 5*s* with the XGB model, and 0.8*s* with the SVM model without SFFS on the computer used to run the experiments. However, one iteration with SVM model when SFFS was used took on average ~7.7 min, so SFFS added a significant computational load to model training.

Classification experiments were conducted in Python. The *xgboost* package was used for XGB implementation and the *scikit-learn* implementation for other classifiers. Bayesian optimization was conducted with the *hyperopt* and SFFS feature selection with the *mlxtend* packages.

#### 2.4.2 Model explanations

The paradigm of explainable artificial intelligence (XAI), which aims to help understand how opaque ML models work, has been acknowledged as a crucial feature for practical ML models in recent years (Barredo Arrieta et al., 2020). In this work, XAI was applied to the ML models using feature relevance explanation techniques that described the functioning of the model by measuring the importance each feature had to the prediction output. The importance of features for classification was assessed with Shapley additive explanations (SHAP).

SHAP is a local, model-agnostic explanation method based on game theory. It has a solid mathematical background with desirable properties for an explanation method, which are discussed thoroughly in Lundberg and Lee (2017). As a local method, SHAP values are computed for each observation. To draw conclusions on global model behavior, summary statistics or visualizations are needed. A single SHAP value describes the effect that an observed feature value has on the model output as compared to average prediction (Molnar, 2022). So, in the case of classification, a SHAP value of e.g., 0.04 of a feature with respect to a given class denotes that the probability of being classified to the given class was 4%-points higher than it was on average. The global importance of each feature can be estimated as an average absolute SHAP value, and investigation of the distribution of individual SHAP values in relation to feature values allows assessing how changes in feature values are related to changes in model output.

## 3 Results

### 3.1 Mixed model results

The physiological stress reactions during the CPT and MA trials of the MAST were studied with linear mixed model analysis. The results showed statistically significant effect of stress type (BL, CPT, MA) on all features, expect for blink skewness and the time between saccades. The residual normality was verified from the histograms and q-q plots for all the models with statistically significant effect for the stress type. *Post-hoc* analyses of all the significant features are presented in Table 3. Detailed results of the mixed model analysis including analysis of variance model comparison, models fixed and random effects as well as effect sizes are included in Supplementary material.

**Table 3:** Results of the mixed model *post-hoc* analyses. The direction of the effect can be deduced from the sign of the *Z*-value.

	**CPT-BL**		**MA-BL**		**MA-CPT**	
**Feature**	***z*** **-value**	***p*** **-value**	***z*** **-value**	***p*** **-value**	***z*** **-value**	***p*** **-value**
ecg_hr_mean	13.14	< 0.001	25.31	< 0.001	14.47	< 0.001
ecg_hrv_ibi_mean	−14.04	< 0.001	−25.21	< 0.001	−13.58	< 0.001
ecg_hrv_rmssd	−3.15	0.005	−7.22	< 0.001	−4.66	< 0.001
eog_blink_br_mean	10.13	< 0.001	17.14	< 0.001	8.73	< 0.001
eog_blink_tbb_mean	−9.89	< 0.001	−10.79	< 0.001	−2.37	0.046
eog_blink_bdur_mean	5.01	< 0.001	0.69	0.768	−3.73	< 0.001
eog_sac_sr_mean	1.20	0.454	3.72	< 0.001	2.78	0.0148
eog_sac_sdur_mean	16.36	< 0.001	17.75	< 0.001	3.84	< 0.001
eda_npeaks	0.31	0.949	8.75	< 0.001	8.80	< 0.001
eda_mean	4.23	< 0.001	19.45	< 0.001	16.30	< 0.001
brainbeat	−0.25	0.966	6.00	< 0.001	6.37	< 0.001

The Bonferroni corrected significance level for *p*-values in these tests was 0.005.

### 3.2 Classification results

#### 3.2.1 Overall performance

Longer windows tended to perform better regardless of classifier type and feature set. The best balanced accuracy at a 45 *s* window length was 86.5%, which was clearly higher than the best at a 10*s* window length, 74.8%. The best single signal seemed to be EOG for almost all classifiers and window durations but feature sets with multiple signals performed better than single signals. The combined ECGEDAEOGBB performed the best out of all feature sets across all classifiers, except for QDA.

**Figure 2:**
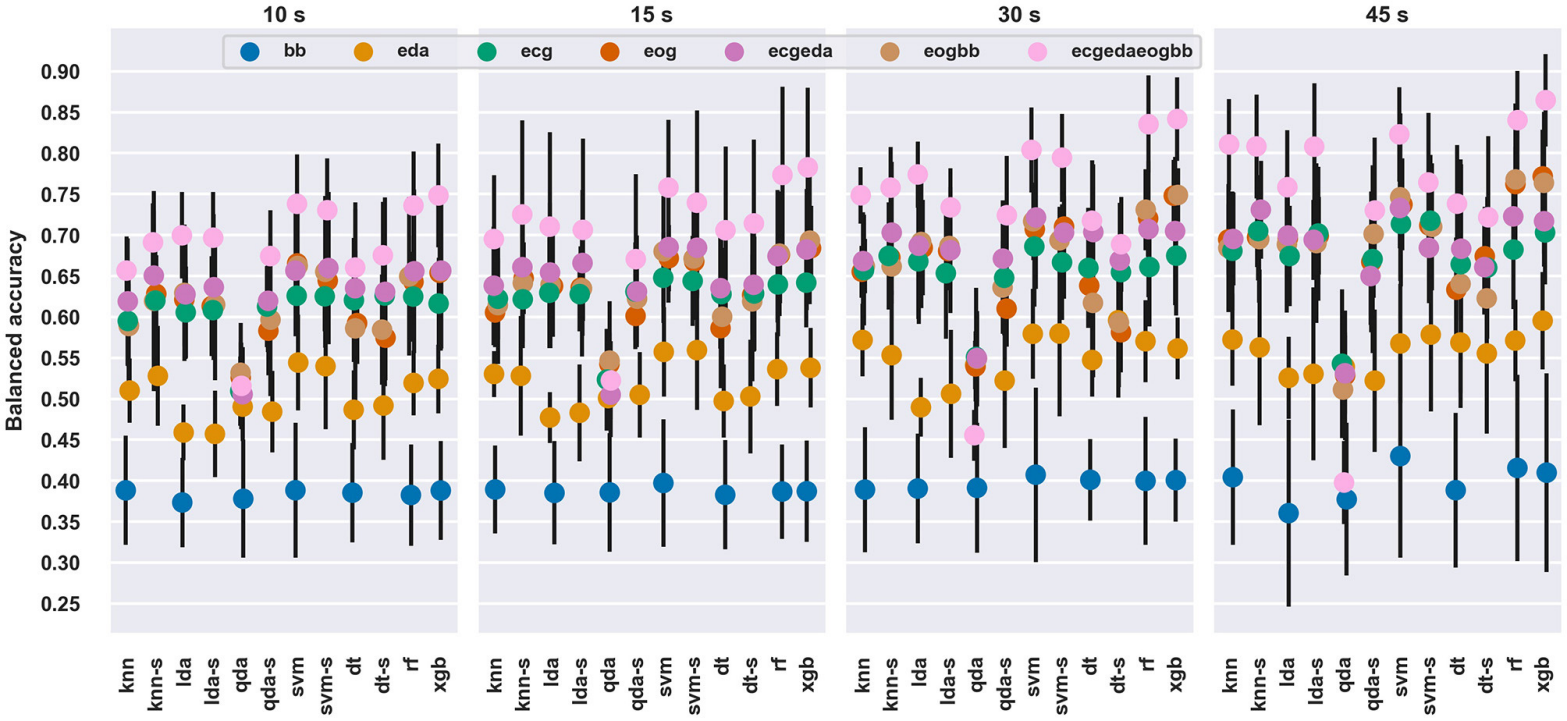
Average balanced accuracy and standard deviation across the window lengths, feature sets and classifiers. The average and standard deviation were computed across the validation folds. The “-s” after a model name denotes scores with SFFS feature selection.

SVM provided the best performance with 12 out of 28 window length and feature set combinations, followed by XGB (10), SVM with SFFS (3), DT with SFFS (2), and RF (1). The balanced accuracies for each set of features with each classifier and each window length are shown in Figure 2. For a deeper investigation, the classification results are available in numerical format as Supplementary material.

The differences between classifiers or feature sets were modest: average performances were usually within standard deviations, except for the BB feature set and QDA classifier. This is understandable, since the BB feature set consisted of only a single feature, as opposed to multiple features in other feature sets. For most window durations and signals, QDA classifier performed poorly, which likely occurred due to its sensitivity to multicollinearity, as e.g., HRV variables tend to be correlated. When SFFS was run with QDA, it performed similarly to other classifiers.

Table 4 shows the confusion matrices for each window duration for the XGB model with ECGEDAEOGBB feature set. Predicting baseline when the actual observations were from a stressful state was uncommon: 3.9 − 5.6% and 0.9 − 1.8% of observations from CPT and MA, respectively, were classified as BL at each window length. Similarly, the amount of BL data classified as MA was between 0.4 and 4.2%. Instead, the two stressful states were increasingly confused with each other when window duration decreased, and similarly predicting CPT instead of BL was increasingly more common with shorter windows.

**Table 4:** Confusion matrices of the XGB model for each window length with all available features, values as percentages of true labels.

	**Predicted label**						
		**BL**	**MA**	**CPT**	**BL**	**MA**	**CPT**
True label			45 *s*			30*s*		
	BL	89.9	0.4	9.7	85.8	1.0	13.2
	MA	1.6	80.6	17.8	1.8	82.4	15.8
	CPT	3.9	7.2	88.9	5.6	10.1	84.2
		15*s*			10*s*		
	BL	76.3	2.9	20.8	71.5	4.2	24.4
	MA	1.7	76.8	21.5	0.9	73.5	25.6
	CPT	4.4	13.6	82.0	4.7	16.4	78.9

Therefore, the classifier performed well when the task was to distinguish BL and stress or BL and MA from each other, while classification between the two stressful states was more difficult. Detecting CPT from BL was also more challenging than detecting MA from BL, as CPT exhibited a closer physiological proximity to BL than MA. Similar phenomenon was observed earlier in Tervonen et al. (2021b).

#### 3.2.2 Parameter optimization

**Figure 3:**
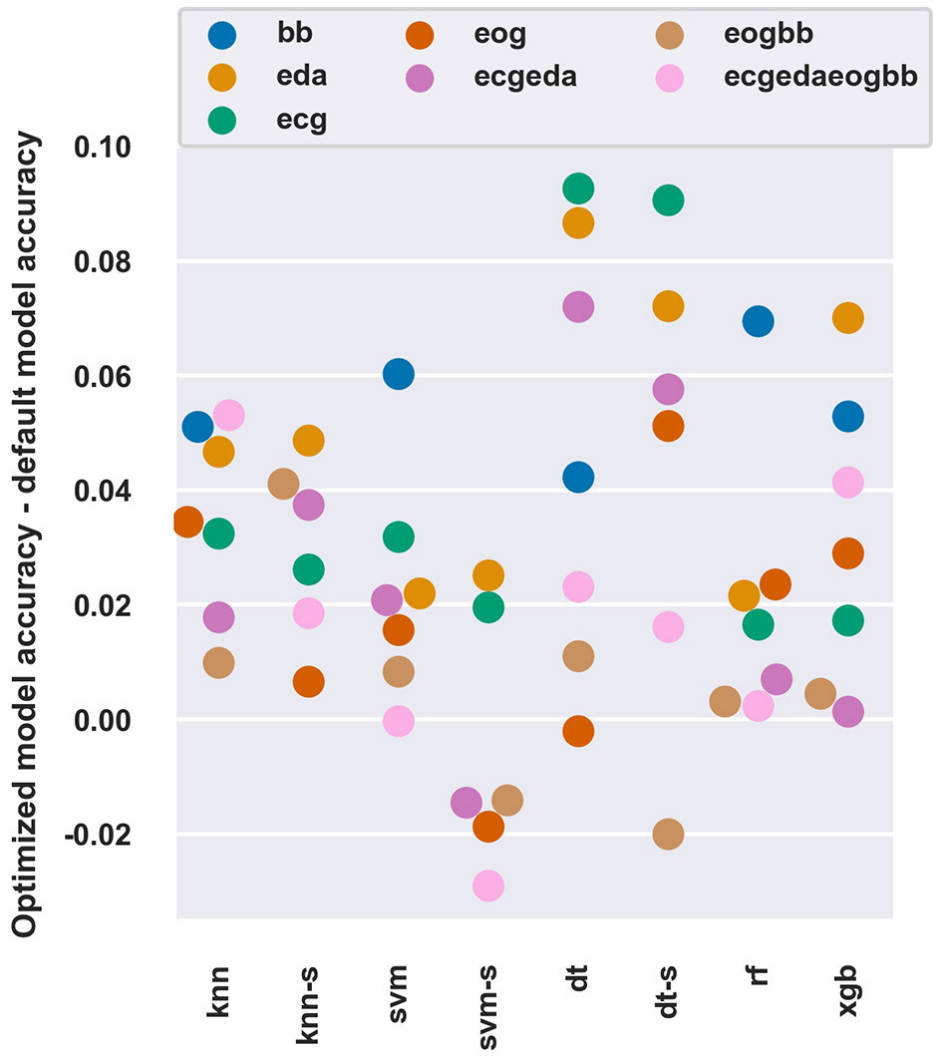
The difference of average balanced accuracy between optimized and default hyperparameters with all models and feature sets at a 45 *s* window length. The “-s” after a model name denotes use of SFFS feature selection.

Figure 3 shows how beneficial Bayesian hyperparameter optimization was as compared to using default hyperparameters (as determined for each classifier in each of the software packages used) with the 45 *s* window length.

The optimization improved balanced accuracy by 1 − 6 percentage points for most models and feature sets. The DT algorithm seemed to benefit the most with up to 9.1 percentage point improvement. Most of the improvements occurred within the first iterations, and on average further improvements were limited to < 1 percentage point after ten iterations of the Bayesian optimization algorithm (data not shown).

Overall, feature sets with lower performance (e.g., BB and EDA), which also had more room for improvement, seemed to benefit more from parameter optimization than feature sets with higher performance (e.g., EOG). Interestingly, the optimization process could not discover hyperparameters that performed better than the default ones in some conditions (e.g., SVM-SFFS with ECGEDAEOGBB features). These observations are in line with Tervonen et al. (2021a) who reported a 3.7 percentage points increase in binary workload classification accuracy, and in general, with most datasets the hyperparameter optimization yields little or no improvement (Sun and Pfahringer, 2013; Tran et al., 2020).

#### 3.2.3 Explainability analysis

The focus on this section is on the best performing classifier, XGB with 45 *s* window length and ECGEDAEOGBB feature set. Please refer to Table 1 for naming of features.

**Figure 4:**
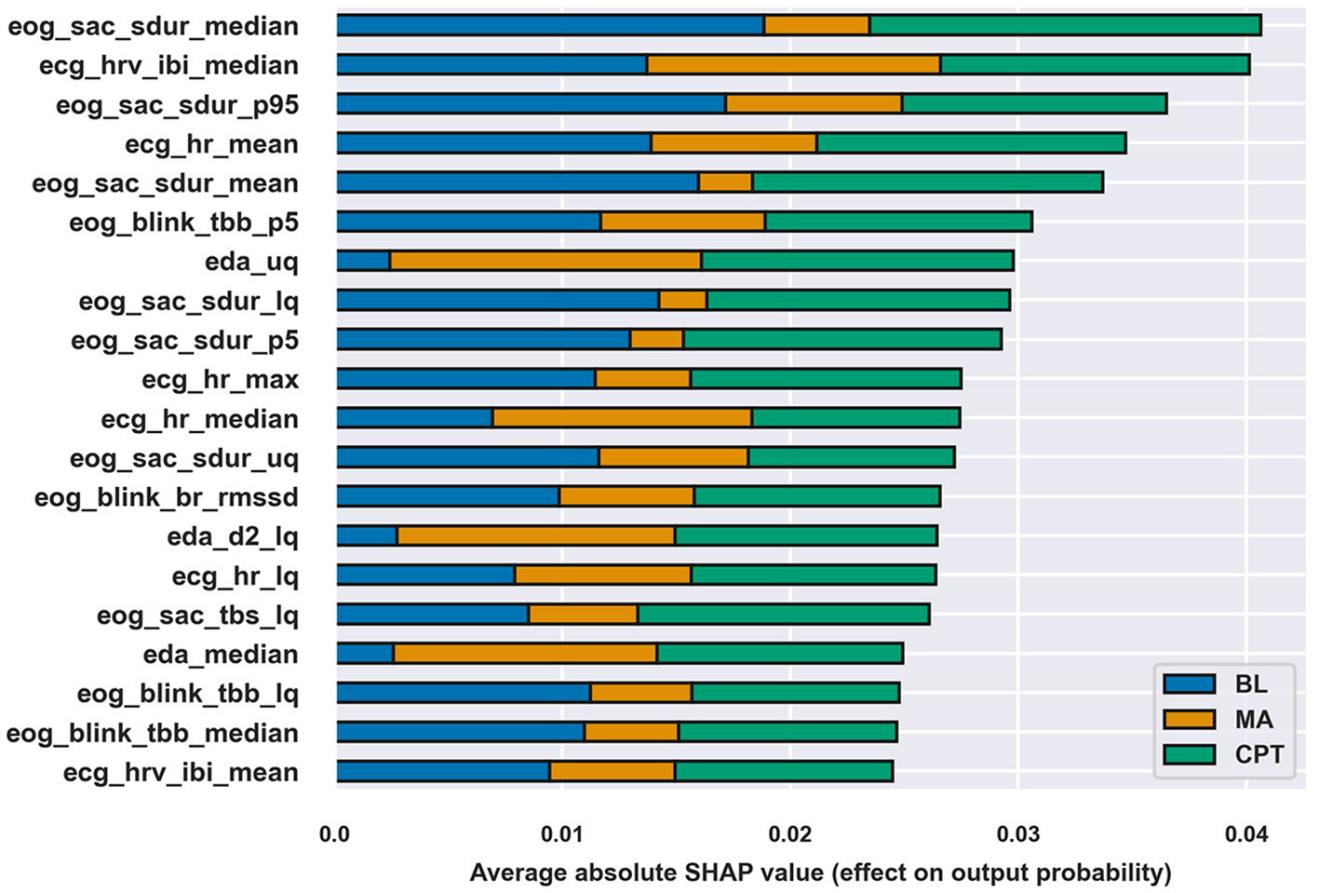
Top-20 features according to absolute SHAP values of the XGB classifier at a 45 *s* window length and the feature set ECGEDAEOGBB. The length of the bar for each class describes the class-wise importance of the feature.

Evaluating feature importance as absolute SHAP value for each class allows analyzing the impact each feature had on separating the class from others. Figure 4 shows top-20 most important features according to absolute SHAP values. The most influential features were related to saccade duration (six features) and all signals except for BB had some features in the top-20. The differences between the total importance of features were modest, but the figure shows that e.g., features on saccade duration were especially important for the correct classification of BL and CPT, while EDA features were more involved in distinguishing MA.

**Figure 5:**
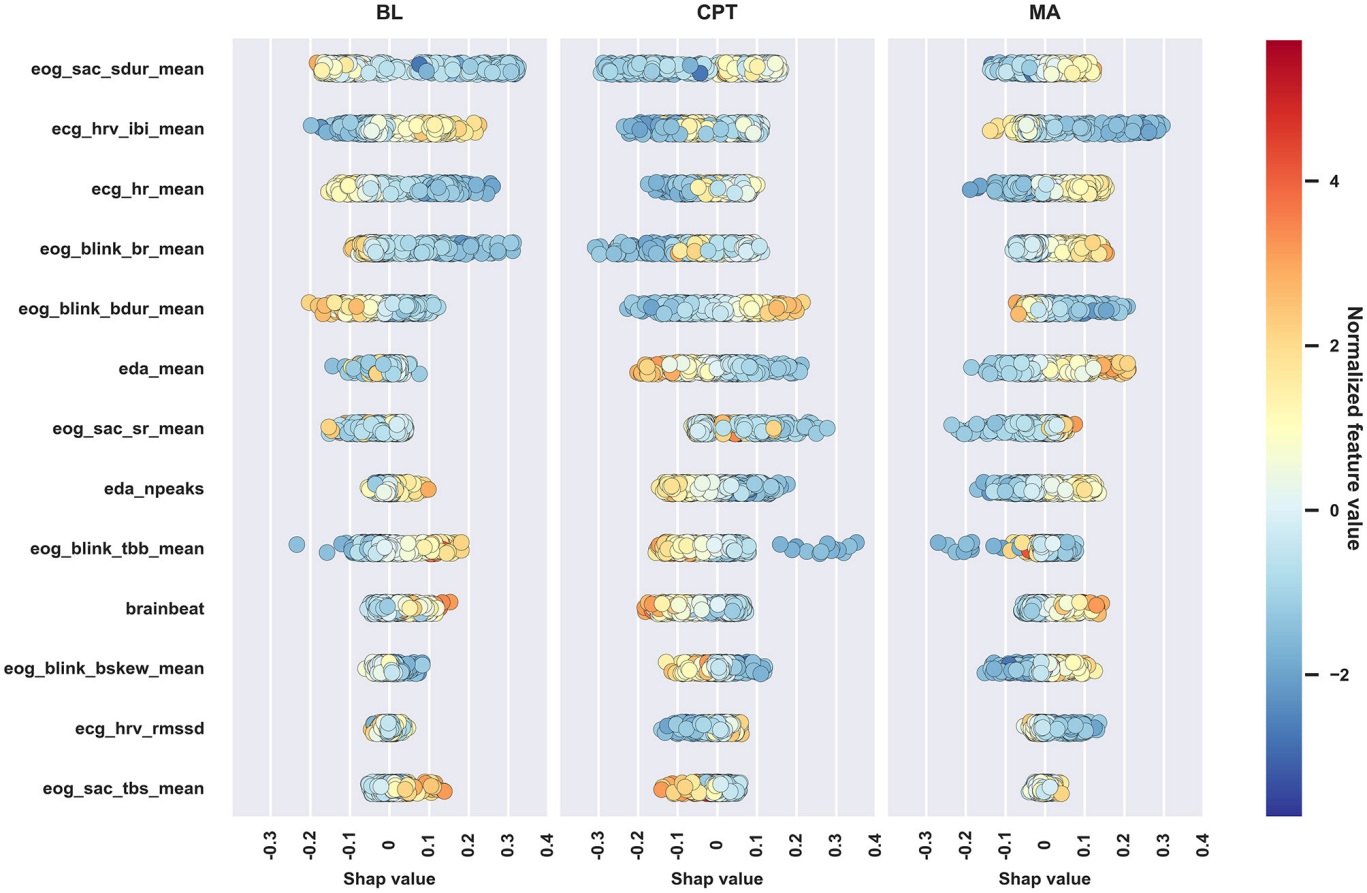
Beeswarm plot of the SHAP values of the interpretable feature set. Markers correspond to observations in the data. The marker colors reflect feature values and the location on the horizontal axis shows how that feature affects the probability of being classified into each of the three classes. Features are ordered according to absolute SHAP value, descending.

However, absolute SHAP values show no direction of the contributions of different features and many of the features are difficult to interpret. Therefore, the inspection is continued with the interpretable set of features defined in Section 2.2. First, classification was redone with the XGB model at 45 *s* window length. After optimizing hyperparameters, the balanced accuracy with the interpretable feature set was 81.5%, which is rather similar to the full set of features, 86.5%, considering that only 8.7% of features were used. So, simplifying the model by reducing the number of features by a large margin and by using features that are easier to understand led only to slightly lower performance. Following the classification, SHAP values were computed and a beeswarm plot of the SHAP values was drawn in Figure 5 to more carefully assess the ways in which features affected classification, which is discussed next.

## 4 Discussion

### 4.1 Physiology, feature importance and contributions to classification

Figure 5 supports the mixed model finding that physiological reactions between the stress types are different, and for nearly all features, the direction of the impact is similar to the direction of the effect in the mixed models; Table 5 summarizes the findings. The discrepancies between mixed model and SHAP results occur since the methods are overall different, SHAP (through the XGB classifier) also takes feature correlations into account, and despite that some features may not show statistically significant difference between conditions (i.e., show no difference in mixed models), they may differ enough to improve classification which displays in the SHAP values.

**Table 5:** Summary of the effects of CPT and MA to the interpretable features as compared to BL.

**Feature**	**Mixed models**	**SHAP**
ecg_hr_mean	Increased in both	Agrees, but less obvious in CPT
ecg_hrv_ibi_mean	Decreased in both	Agrees, but less obvious in CPT
ecg_hrv_rmssd	Decreased in both	Agrees in MA
eda_mean	Increased in both	Agrees in MA
eda_npeaks	Increased in MA	Agrees in MA
eog_blink_br_mean	Increased in both	Agrees, but less obvious in CPT
eog_blink_bdur_mean	Increased in CPT	Agrees in CPT, but decreased in MA
eog_blink_tbb_mean	Decreased in both	Agrees in CPT, but less obvious in MA
eog_blink_bskew_mean	No difference	Increased in MA, decreased in CPT
eog_sac_sr_mean	Increased in MA	Agrees in MA, but decreased in CPT
eog_sac_sdur_mean	Increased in both	Agrees
eog_sac_tbs_mean	No difference	Small effect, decreased in CPT
brainbeat	Increased in MA	Agrees in MA, decreased in CPT

The traditional stress-related markers such as HR and HRV parameters and mean EDA suggest that MA caused stronger effects than the CPT and BL, while the CPT caused slightly more stress than BL. Despite that the reactions varied between the subtasks, the current study expresses no verification for whether MA and CPT elicited different types of stress solely through either the HPA or the SAM, as justified in the introduction. Next, physiological reactions are presented and discussed in more detail signal by signal.

#### 4.1.1 Cardiac responses: ECG

HR and its inverse, mean of IBIs, were the most important features of the ECG parameters. As expected, HR was lower at BL and the psychosocial stressor (MA) increased it, which shows in both Table 3 and Figure 5. According to the mixed model results, HR increased during CPT as compared to BL, but the direction of the HR change in CPT was less obvious in Figure 5. The behavior may follow from three reasons: (1) the features were normalized to have zero mean on an individual level in the ML analysis, (2) feature interactions affected the results and thus the SHAP values in the ML analysis, and (3) features were considered separately without normalization in the mixed model analysis. As seen from Figure 5, higher HR than one's own average increased the chance of being classified to CPT, and lower HR, which is the case for majority of data during CPT, decreased this chance. The result may also reflect individual differences in HR reactions, as Mourot et al. (2008) reported. They found that some of the participants showed an increasing HR trend during the 3-min CPT, while the other participants' HR started to decrease after a short initial increase. Sendowski et al. (1997) reported that HR began to decrease after one minute of cold immersion. In the current study, little of the CPT data come from the condition where CPT has lasted for over a minute.

HRV, characterized by parameter RMSSD, classically indicates stress (Schmidt et al., 2019): stress attenuates HRV. Interestingly, the role of RMSSD was clear but quite modest, both in mixed models (Table 3) and in SHAPs (Figure 5), indicating that in this protocol, CPT was less stressful than MA. According to mixed models, RMSSD was lower during MA than CPT and according to the SHAP values, lower RMSSD values were associated with lower chance of being classified to CPT and higher chance of being classified to MA.

#### 4.1.2 Eye parameters: EOG

The saccade duration links directly to the magnitude of the eye movement, the longer the duration the larger the eye movement. The saccade duration contributed strongly to all conditions, especially in BL and CPT. Brief saccades associated with BL and longer ones with the stress states. One reason for this distinction may be related to the experimental setup, as the visual information differed between BL and MAST. During the BL, participants kept their gaze on a fixation point in the middle of a screen, whereas in both MAST trials the instructions (two lines of text) were visible on the screen. Although the visual stimulus matched between CPT and MA, the trials exhibited a clear disparity: the participants made shorter saccades in CPT. This means that the saccade duration may also contain information about the type of stress and not just the visual stimulus.

Saccade rate (SR) reflects eye activity: the higher the SR the more actively the person scans the environment (e.g., visual search). According to the mixed model analysis, the participants moved their eyes more often in MA than in BL and CPT, which is also visible in Figure 5: higher SR contributes to MA classification whereas its contribution in BL and CPT is confusing. In MA, the seed number was visible on the screen and the participants may have scanned the number while performing mental calculation, which may increase SR compared to BL and CPT.

Blink rate (BR) and time between blinks (TBB) both reflect how actively a person blinks. MA induced the highest BR, but also CPT increased the BR compared to BL. In earlier studies, CPT and painful stimulation in general have increased the BR (Paparella et al., 2020). Attentional engagement decreases the number of blinks during cognitively demanding tasks that require visual attention (Maffei and Angrilli, 2019; Ranti et al., 2020). Here MA was cognitively demanding but required no visual attention. Similar MA tasks have increased the BR and giving answer verbally has increased BR significantly compared to a non-verbal condition (Schuri and von Cramon, 1981). Moreover, MA in this study contained time pressure and penalization exposing participants to a social-evaluative threat. This uncomfortable situation might have caused anxiety or even fear, which also increases BR (Maffei and Angrilli, 2019).

Blink duration, i.e., the time the eye is closed while blinking, contributes positively to CPT (longer blinks) and negatively to BL and MA (shorter blinks). Increased blink duration indicates sleepiness (Schleicher et al., 2008), however, decreased vigilance increases especially the re-opening time of the eye, which in turn skews the blink's waveform (blink skewness). Here the blink skewness showed no changes in different stress stages, suggesting that the blink duration may be a sufficient indicator of the discomfort and even pain experienced during the cold pressor. However, no literature was found addressing the link between blink duration and discomfort or pain.

#### 4.1.3 Skin conductivity response: EDA

The amplitude of the phasic EDA increased in both stressor tasks (Table 3), but the effect was stronger in MA, indicating higher stress, and MA and CPT indeed differed from each other significantly. The same was seen even more clearly in the number of EDA peaks: there was no difference between CPT and BL but both were different from MA. These observations are in line with SHAP values in Figure 5. Higher values in phasic EDA and number of peaks were related to higher chance of being classified into MA. Consequently, higher values decreased the chance of being classified into BL and CPT and lower values increased this chance.

EDA activity is linked to the arousal level and activation of the sympathetic ANS branch, suggesting that CPT causes slightly larger sympathetic activation than BL, and MA clearly larger than CPT. Recently, Lee (2021) provided a comprehensive summary of the connection between EDA and engagement, attention, and alertness: increased EDA was linked to e.g., cognitive effort during solving arithmetic problems or reading a map, and state anxiety while being asked to give a public unplanned presentation. This agrees with the results in this study: EDA response is strong in MA, demanding cognitive effort and attention. EDA has also been used in assessment of pain perception and the feasibility of an EDA-based algesimeter was introduced in Storm (2008) with promising results. On a group level, EDA appears to be less sensitive pain marker than HR, yet on an individual level, EDA correlates with pain ratings better than HR (Loggia et al., 2011). However, in the MAST protocol, the induced pain may not be comparable to stimuli used in actual pain studies and care should be used in comparing the results.

#### 4.1.4 Cognitive load: EEG

Brainbeat indicates the level of cognitive loading and attention (Holm et al., 2009). Mixed model analysis (Table 3) accentuated that only the MA increases the BB. According to Figure 5, BB had low feature importance, but its behavior was curiously different from other features. High BB values related to higher chance of being classified to BL and MA, and low BB values to CPT. MA challenges one cognitively, but BL is supposed to be a relaxing and even boring phase of the protocol, which, when looking at HR, HRV and EDA, seems to be so. However, the brain seems to remain relatively active during BL, whereas during CPT, the cognitive load seems to diminish, possibly as a recovery reaction following the high-load MA phase. In the CPT phase, the participants mentally relax from the MA, which is also visible in RMSSD, even if the CPT itself is physically uncomfortable. Interestingly, many EEG studies of pain have used CPT as pain induction method (Chang et al., 2002; Chouchou et al., 2021), and those studies have found increased frontal theta and decreased parietal alpha activity in pain condition, i.e., effects that would show as increased BB. In those experiments, the CPT duration has typically been longer, cumulating the pain sensation, and not preceeded by a cognitively stressful phase.

### 4.2 Characteristics and consequences of the stress induction protocol

When comparing the MAST results to results from protocols inducing only one type of stress, there are some differences to be considered. The trials in MAST last from 45 to 90*s*. In protocols employing stressors with longer durations, adaptation and habituation may contribute more. On the other hand, in MAST, repetition introduces a learning effect, especially in MA. As MAST has no recovery delay between MA and CPT phases, the physiological adaptation to the new stressor will take some time, and the response time will depend on the biosignal. The adaptation time, or physiological leakage, makes classification more challenging, especially during the first windows, which may be erroneously classified into the previous subtask. The dynamics of the physiological signals during MAST as time series is an obvious research need arising from the current work.

The CPT subtasks in MAST last for at most 90*s* and the hand is warmed up by a heatpad during the interleaved MA trials. Compared to pain induction protocols where the maximum pain rating is typically reached at around 90 s through the 3-min CPT (Chang et al., 2002), the average pain in MAST is likely lower than in a standard pain-induction CPT. Moreover, the CPT in this study was applied to the right (dominant) hand, for practical purposes, as the left hand was kept immobile to ensure good signal in EDA, and the dominant hand was needed to operate the questionnaires. McGinley and Friedman (2015) have indicated that CPT would produce larger effects when applied to the left hand. This indeed could have produced even stronger responses and in protocols where the target hand can be selected freely, the left one should be favored.

### 4.3 Machine learning pipeline components

Table 6 presents a summary of the effect of different ML components and learnings from them. The effect of each component was assessed as difference in average balanced accuracy across all the other components. The average applied was harmonic mean, since the average was taken over relative values (balanced accuracies). Overall, the selection of the signals to measure and to use for classification showed the largest effect, whereas the other choices resulted with moderate to low effect.

**Table 6:** Observations made from the investigated ML pipeline components, and their effect on balanced accuracy.

**Choice**	**Observations**	**Comparison**	**Effect**
Signals to measure	- EOG and ECG were the best single signals	- to EDA and to BB	+0.11, +0.25
	- Adding signals improved performance	- ECGEDAEOGBB to EOG	+0.06
	- Best two signal combination was ECGEDA	- to EOGBB	+0.01
Length of feature windows	- Longer windows performed better	- 45 *s* window to 10*s* window	+0.05
Feature selection	- SFFS was useful but at high computational cost	- models with vs. without SFFS	+0.07
	- Interpretable set of features based on SHAP values performed nearly as well as the full set	- Interpretable to full feature set with XGB and 45 *s* window	−0.05
Classifier	- XGB showed highest performance	- to the next best model RF	+0.02
Hyperparameter optimization	- Benefit rather limited	- optimized to default parameters	+0.02^*^
	- Few iterations of Bayesian optimization suffices	−50 vs. 5 iterations	+0.003^*^
	- Models and feature sets with poorer performance seemed to benefit more	- see Figures 2, 3	-

Effect values are harmonic means of balanced accuracies obtained across the other choices.

^*^Arithmetic mean, since some values were negative.

Therefore, practitioners and researchers should pay special attention to the choice of the signals they measure and consider the viability of measuring several for their application. Once the signals have been selected, using longer feature windows or selecting features with SFFS seem to be the best alternatives. However, SFFS comes with a notable computational load, which may hinder its applicability especially with larger datasets. The choice of the duration of feature windows was limited to at most 45 *s* in this study because of the data collection protocol. Short windows enable closer-to-real-time state detection, but the performance decreased as window duration decreased. The overall impression has been noted before (Gjoreski et al., 2017; Siirtola, 2019; Tervonen et al., 2021a). This likely happens since shorter feature windows enclose less data and thus, less information, and less physiological events than longer ones. In addition, shorter windows contain fewer samples and consequently, the contribution of noise and transient artifacts is higher than in longer windows. Considering that the 10*s* window contained almost five times less data than the 45 *s* window but still approximately three out of four classifications were correct, shorter windows may have some use in applications where more timely state detection is necessary, such as driver monitoring. In some other domains, like chronic stress management, hourly or even daily stress detection may be sufficient. Thus, the selection of window duration comes down to requirements of the application under investigation, which should be considered when interpreting the results in this paper.

Reducing the number of features decreased model performance by 5 percentage points. However, such reduction allowed to study the features effects on classification and reflect them to human physiology (see Section 4.1). This examination showed that the model concentrated on physiologically relevant phenomena. Such analysis gives the model some ecological validity and helps build trust to it. Had it been conducted with the full set of 150 features, making conclusions at such detailed level would have been overwhelming. Therefore, opting for fewer features in favor of interpretability is desirable in any application that requires trust to the ML model.

### 4.4 Limitations and future work

The sample size in the dataset was rather small, but comparable to sample sizes documented in several review articles: Giannakakis et al. (2022) reported an average of 23 participants in stress detection studies, Schmidt et al. (2019) an average of 21 participants in stress and affect detection studies, and Vos et al. (2023) conveyed an average sample size of 21 in open stress detection datasets, and an average number of participants of 19 in stress detection studies. As noted in Vos et al. (2023), the problem of small sample sizes is common in machine learning based stress detection research.

The critique extends to this research. Moreover, the sample analyzed comprised young and healthy people and the measurement setup was too heavy to incorporate in real-life field studies. However, as an explorative study, a homogeneous sample helps diminish the effects of different confounding factors, and the main goals were to determine whether the reactions in ECG, EDA, EOG, and BB deviated between the baseline and stress types and whether a ML model could detect the stress types from baseline. The results indicate sufficiently positive outcome to justify future studies with a larger and more diverse sample, or potentially with several datasets to increase sample size and facilitate cross-dataset comparisons. As the features employed for classification can also be extracted from light-weight wearable devices, semi-constrained and even real-life studies with several types or levels of stress and more dynamic state changes are viable.

The dynamicity posed a challenge already in this study, as the MAST protocol lacks rest periods between the two stressor tasks. This likely caused some physiological leakage when the task changed, affecting classification performance. As resting and recovering after each stressor is impossible in real-life, detection methodologies must be able to handle such complication, and future studies should with several stress types would benefit from a close inspection of the amount of leakage and its implications to classification.

In the current study, missing data exhibited mainly in EOG features in windows with few or no blinks conducted and in windows with too few or too small saccades, or too noisy signal to extract saccades. Windows with missing values were dropped after feature fusion to allow the models to benefit from all available data. This choice, however, caused some variation in the number of samples per feature set at each window length, displayed in Table 2. Dropping the values before feature fusion improved classification performance on average by 0.6%, so the effect of missing data handling was rather small. Since missing values are bound to occur either randomly due to e.g., sensor malfunction or systematically due to not making any blinks, handling these cases carefully is important for robust stress detection in the field. Potential future solutions include imputation and maximum likelihood methods, or the detection could rely on single signal models when data from one signal is missing.

Finally, stress reactions are subjective. The current study acknowledged this only by normalizing feature separately for each participant before classification. While the approach is common and shown to be effective, it requires a full set of data from each participant, and thus it is infeasible in application which expect new users: features could not be normalized for the new person until sufficient data was collected. Furthermore, the participant-wise balanced accuracy varied from 56 to 100% for the most accurate model, and so the model performed poorly for some people. It could be that those persons were indeed not stressed, but to improve the performance and the practical value of the model, future work could consider personalization approaches that improve the performance for the most difficult participants and operate on minimal data.

## 5 Conclusions

A set of physiological signals was successfully used to distinguish two stress conditions from each other and from a baseline condition, both using a mixed model analysis and applying ML methods. Mixed model analysis found that physiological responses to the different stressors varied and ML models could classify the stressor tasks and baseline with decent performance. The two analysis methods agreed well on the importance and direction of the effect of various physiological features, and these findings were in line with the existing literature on stress psychophysiology. The contribution of features reflecting ECG and EOG outweighed the impact of EDA and brainbeat (EEG).

In stress type classification using ML, signal windowing, feature selection, classifier choice, and hyperparameter optimization attenuated in favor of the choice of the biosignals and computed features. The work demonstrated that stress type detection was feasible using measurements that can be implemented on wearable devices. The best accuracy was achieved using a multimodal set of biosignals and features. Subsequently, the limited input information could not be compensated by choices made in the data analysis.

As the first investigation to the divergent physiological reactions within the MAST, the study provides a basis for near real-time acute stress type detection with dynamic state changes. However, larger cross-dataset comparisons are needed before applying it in real-life use cases.

## Data availability statement

The dataset analyzed in the study cannot be made available due to ethical reasons as permission was not obtained from the study participants.

## Ethics statement

The studies involving humans were approved by Ethics Committee in the Humanities and Social and Behavioural Sciences of the University of Helsinki. The studies were conducted in accordance with the local legislation and institutional requirements. The participants provided their written informed consent to participate in this study.

## Author contributions

JT: Conceptualization, Formal analysis, Investigation, Methodology, Software, Validation, Visualization, Writing – original draft, Writing – review & editing. JN: Conceptualization, Data curation, Funding acquisition, Investigation, Resources, Writing – original draft, Writing – review & editing. JM: Conceptualization, Project administration, Resources, Supervision, Writing – review & editing. KP: Conceptualization, Formal analysis, Funding acquisition, Investigation, Methodology, Software, Validation, Visualization, Writing – original draft, Writing – review & editing.

## References

[B1] Aristizabal S. Byun K. Wood N. Mullan A. F. Porter P. M. Campanella C.. (2021). The feasibility of wearable and self-report stress detection measures in a semi-controlled lab environment. IEEE Access 9, 102053–102068. 10.1109/ACCESS.2021.3097038

[B2] Barredo Arrieta A. Díaz-Rodríguez N. Del Ser J. Bennetot A. Tabik S. Barbado A.. (2020). Explainable artificial intelligence (xai): concepts, taxonomies, opportunities and challenges toward responsible ai. Inf. Fusion 58, 82–115. 10.1016/j.inffus.2019.12.012

[B3] Bates D. Mächler M. Bolker B. Walker S. (2015). Fitting linear mixed-effects models using lme4. J. Statist. Softw. 67, 1–48. 10.18637/jss.v067.i01

[B4] Benedek M. Kaernbach C. (2010). A continuous measure of phasic electrodermal activity. J. Neurosci. Methods 190, 80–91. 10.1016/j.jneumeth.2010.04.028 20451556 PMC2892750

[B5] Bentivoglio A. R. Bressman S. B. Cassetta E. Carretta D. Tonali P. Albanese A. (1997). Analysis of blink rate patterns in normal subjects. Mov. Disord. 12, 1028–1034. 10.1002/mds.870120629 9399231

[B6] Bergstra J. Yamins D. Cox D. (2013). “Making a science of model search: hyperparameter optimization in hundreds of dimensions for vision architectures,” in Proceedings of the 30th International Conference on Machine Learning, Volume 28 of Proceedings of Machine Learning Research, eds S. Dasgupta, and D. McAllester (Atlanta, GA: PMLR), 115–123.

[B7] Chalabianloo N. Can Y. S. Umair M. Sas C. Ersoy C. (2022). Application level performance evaluation of wearable devices for stress classification with explainable ai. Pervasive Mob. Comput. 87, 101703. 10.1016/j.pmcj.2022.101703

[B8] Champseix R. (2018). Heart Rate Variability Analysis. Available online at: https://github.com/Aura-healthcare/hrv-analysis (accessed September 14, 2023).

[B9] Chang P. F. Arendt-Nielsen L. Chen A. C. (2002). Dynamic changes and spatial correlation of eeg activities during cold pressor test in man. Brain Res. Bull. 57, 667–675. 10.1016/S0361-9230(01)00763-8 11927371

[B10] Chouchou F. Perchet C. Garcia-Larrea L. (2021). Eeg changes reflecting pain: is alpha suppression better than gamma enhancement? Neurophysiol. Clin. 51, 209–218. 10.1016/j.neucli.2021.03.001 33741256

[B11] Daniels J. Georgiou P. (2019). “A data-driven detection system for predicting stress levels from autonomic signals,” in 2019 IEEE Biomedical Circuits and Systems Conference (BioCAS) (Nara), 1–4.

[B12] Dickerson S. S. Kemeny M. E. (2004). Acute stressors and cortisol responses: a theoretical integration and synthesis of laboratory research. Psychol. Bull. 130, 355–391. 10.1037/0033-2909.130.3.355 15122924

[B13] Feng M. Fang T. He C. Li M. Liu J. (2023). Affect and stress detection based on feature fusion of lstm and 1dcnn. Comp. Methods Biomech. Biomed. Eng. 0, 1–9. 10.1080/10255842.2023.2188988 36919485

[B14] Ghiasi S. Greco A. Barbieri R. Scilingo E. P. Valenza G. (2020). Assessing autonomic function from electrodermal activity and heart rate variability during cold-pressor test and emotional challenge. Sci. Rep. 10, 5406. 10.1038/s41598-020-62225-2 32214158 PMC7096472

[B15] Giannakakis G. Grigoriadis D. Giannakaki K. Simantiraki O. Roniotis A. Tsiknakis M. (2022). Review on psychological stress detection using biosignals. IEEE Transact. Affect. Comp. 13, 440–460. 10.1109/TAFFC.2019.2927337

[B16] Gjoreski M. Luštrek M. Gams M. Gjoreski H. (2017). Monitoring stress with a wrist device using context. J. Biomed. Inform. 73, 159–170. 10.1016/j.jbi.2017.08.006 28803947

[B17] Gjoreski M. Kolenik T. Knez T. Luštrek M. Gams M. Gjoreski H.. (2020). Datasets for cognitive load inference using wearable sensors and psychological traits. Appl. Sci. 10. 10.3390/app10113843

[B18] Hastie T. Tibshirani R. Friedman J. (2009). The Elements of Statistical Learning. Springer Series in Statistics, 2nd Edm. New York, NY: Springer.

[B19] Hellhammer J. Schubert M. (2012). The physiological response to trier social stress test relates to subjective measures of stress during but not before or after the test. Psychoneuroendocrinology 37, 119–124. 10.1016/j.psyneuen.2011.05.012 21689890

[B20] Henderson J. M. Shinkareva S. V. Wang J. Luke S. G. Olejarczyk J. (2013). Predicting cognitive state from eye movements. PLoS ONE 8, e0064937. 10.1371/journal.pone.0064937 23734228 PMC3666973

[B21] Holm A. Lukander K. Korpela J. Sallinen M. Müller K. M. I. (2009). Estimating brain load from the EEG. Sci. World J. 9, 639–651. 10.1100/tsw.2009.83 19618092 PMC5823228

[B22] Kirschbaum C. Pirke K. M. Hellhammer D. H. (1993). The “trier social stress test”—a tool for investigating psychobiological stress responses in a laboratory setting. Neuropsychobiology 28, 76–81. 10.1159/000119004 8255414

[B23] Lee V. R. (2021). Youth engagement during making: using electrodermal activity data and first-person video to generate evidence-based conjectures. Inf. Learn. Sci. 122, 270–291. 10.1108/ILS-08-2020-0178

[B24] Loggia M. L. Juneau M. Bushnell C. M. (2011). Autonomic responses to heat pain: heart rate, skin conductance, and their relation to verbal ratings and stimulus intensity. Pain 152, 592–598. 10.1016/j.pain.2010.11.032 21215519

[B25] Luke S. G. (2017). Evaluating significance in linear mixed-effects models in r. Behav. Res. Methods 49, 1494–1502. 10.3758/s13428-016-0809-y 27620283

[B26] Lundberg S. M. Lee S.-I. (2017). “A unified approach to interpreting model predictions,” in Proceedings of the 31st International Conference on Neural Information Processing Systems, NIPS'17 (Red Hook, NY: Curran Associates Inc.), 4768–4777.

[B27] Maffei A. Angrilli A. (2019). Spontaneous blink rate as an index of attention and emotion during film clips viewing. Physiol. Behav. 204, 256–263. 10.1016/j.physbeh.2019.02.037 30822434

[B28] Marshall S. P. (2007). Identifying cognitive state from eye metrics. Aviat. Space Environ. Med. 78(5 Suppl.):B165?B175. 17547317

[B29] McGinley J. J. Friedman B. H. (2015). Autonomic responses to lateralized cold pressor and facial cooling tasks. Psychophysiology 52, 416–424. 10.1111/psyp.12332 25250478

[B30] Mishra V. Sen S. Chen G. Hao T. Rogers J. Chen C.-H.. (2020b). “Evaluating the reproducibility of physiological stress detection models,” in Proc. ACM Interact. Mob. Wearable Ubiquitous Technol. (New York, NY). 10.1145/3432220 PMC9523764 36189150

[B31] Mishra V. Pope G. Lord S. Lewia S. Lowens B. Caine K.. (2020a). Continuous detection of physiological stress with commodity hardware. ACM Trans. Comput. Healthcare 1, 1–30. 10.1145/3361562 32832933 PMC7442214

[B32] Molnar C. (2022). Interpretable Machine Learning. 2 Edn. Available online at: https://christophm.github.io/interpretable-ml-book

[B33] Mourot L. Bouhaddi M. Regnard J. (2008). Effects of the cold pressor test on cardiac autonomic control in normal subjects. Physiol. Res. 58, 83–91. 10.33549/physiolres.931360 18198985

[B34] Mozos O. M. Sandulescu V. Andrews S. Ellis D. Bellotto N. Dobrescu R.. (2017). Stress detection using wearable physiological and sociometric sensors. Int. J. Neural Syst. 27, 1650041. 10.1142/S0129065716500416 27440466

[B35] Paparella G. Di Stefano G. Fasolino A. Di Pietro G. Colella D. Truini A.. (2020). Painful stimulation increases spontaneous blink rate in healthy subjects. Sci. Rep. 10, 20014. 10.1038/s41598-020-76804-w 33203984 PMC7672065

[B36] Pettersson K. Tervonen J. Närväinen J. Henttonen P. Määttänen I. Mäntyjärvi J. (2020). “Selecting feature sets and comparing classification methods for cognitive state estimation,” in 2020 IEEE 20th International Conference on Bioinformatics and Bioengineering (BIBE) (Cincinnati, OH), 683–690.

[B37] Pettersson K. Jagadeesan S. Lukander K. Henelius A. Hæggström E. Müller K. (2013). Algorithm for automatic analysis of electro-oculographic data. Biomed. Eng. Online 12. 10.1186/1475-925X-12-110 24160372 PMC3830504

[B38] Pudil P. Novovieova J. Kittler J. (1994). Floating search methods in feature selection. Pattern Recognit. Lett. 15, 365–366. 10.1016/0167-8655(94)90127-9

[B39] Quaedflieg C. Meyer T. van Ruitenbeek P. Smeets T. (2017). Examining habituation and sensitization across repetitive laboratory stress inductions using the MAST. Psychoneuroendocrinology 77, 175–181. 10.1016/j.psyneuen.2016.12.009 28068575

[B40] Ranti C. Jones W. Klin A. Shultz S. (2020). Blink rate patterns provide a reliable measure of individual engagement with scene content. Sci. Rep. 10, 8267. 10.1038/s41598-020-64999-x 32427957 PMC7237680

[B41] Schleicher R. Galley N. Briest S. Galley L. (2008). Blinks and saccades as indicators of fatigue in sleepiness warnings: looking tired? Ergonomics 51, 982–1010. 10.1080/00140130701817062 18568959

[B42] Schmidt P. Reiss A. Dürichen R. Laerhoven K. V. (2019). Wearable-based affect recognition–a review. Sensors 19. 10.3390/s19194079 PMC6806301 31547220

[B43] Schmidt P. Reiss A. Duerichen R. Marberger C. Van Laerhoven K. (2018). “Introducing wesad, a multimodal dataset for wearable stress and affect detection,” in Proceedings of the 20th ACM International Conference on Multimodal Interaction, ICMI '18 (New York, NY: Association for Computing Machinery), 400–408.

[B44] Schuri U. von Cramon D. (1981). Heart rate and blink rate responses during mental arithmetic with and without continuous verbalization of results. Psychophysiology 18, 650–653. 10.1111/j.1469-8986.1981.tb01839.x 7313024

[B45] Sedghamiz H. (2014). Complete Pan-Tompkins Implementation ECG QRS Detector. MATLAB Central, Mathworks. 10.13140/RG.2.2.14202.59841

[B46] Sendowski I. Savourey G. Besnard Y. Bittel J. (1997). Cold induced vasodilatation and cardiovascular responses in humans during cold water immersion of various upper limb areas. Eur. J. Appl. Physiol. Occup. Physiol. 75, 471–477. 10.1007/s004210050191 9202941

[B47] Sharpley C. F. (2009). Neurobiological pathways between chronic stress and depression: dysregulated adaptive mechanisms? Clin. Med. Insights Psychiatry 2. 10.4137/CMPsy.S3658

[B48] Shilton A. L. Laycock R. Crewther S. G. (2017). The maastricht acute stress test (mast): Physiological and subjective responses in anticipation, and post-stress. Front. Psychol. 8, 567. 10.3389/fpsyg.2017.00567 28469586 PMC5395611

[B49] Siirtola P. (2019). “Continuous stress detection using the sensors of commercial smartwatch,” in Adjunct Proceedings of the 2019 ACM International Joint Conference on Pervasive and Ubiquitous Computing and Proceedings of the 2019 ACM International Symposium on Wearable Computers (New York, NY: Association for Computing Machinery), 1198–1201.

[B50] Smeets T. Cornelisse S. Quaedflieg C. W. Meyer T. Jelicic M. Merckelbach H. (2012). Introducing the maastricht acute stress test (mast): a quick and non-invasive approach to elicit robust autonomic and glucocorticoid stress responses. Psychoneuroendocrinology 37, 1998–2008. 10.1016/j.psyneuen.2012.04.012 22608857

[B51] Storm H. (2008). Changes in skin conductance as a tool to monitor nociceptive stimulation and pain. Curr. Opin. Anesthesiol. 21, 796–804. 10.1097/ACO.0b013e3283183fe4 18997532

[B52] Sun Q. Pfahringer B. (2013). Pairwise meta-rules for better meta-learning-based algorithm ranking. Mach. Learn. 93, 141–161. 10.1007/s10994-013-5387-y

[B53] Tervonen J. Närväinen J. Mäntyjärvi J. Pettersson K. (2021b). “In search of harmful stress,” in Adjunct Proceedings of the 2021 ACM International Joint Conference on Pervasive and Ubiquitous Computing and Proceedings of the 2021 ACM International Symposium on Wearable Computers (New York, NY: Association for Computing Machinery), 215–217.

[B54] Tervonen J. Pettersson K. Mäntyjärvi J. (2021a). Ultra-short window length and feature importance analysis for cognitive load detection from wearable sensors. Electronics 10. 10.3390/electronics10050613

[B55] Tran N. Schneider J.-G. Weber I. Qin A. (2020). Hyper-parameter optimization in classification: to-do or not-to-do. Pattern Recognit. 103, 107245. 10.1016/j.patcog.2020.107245

[B56] Vanhollebeke G. De Smet S. De Raedt R. Baeken C. van Mierlo P. Vanderhasselt M.-A. (2022). The neural correlates of psychosocial stress: a systematic review and meta-analysis of spectral analysis eeg studies. Neurobiol. Stress 18, 100452. 10.1016/j.ynstr.2022.100452 35573807 PMC9095895

[B57] Vos G. Trinh K. Sarnyai Z. Rahimi Azghadi M. (2023). Generalizable machine learning for stress monitoring from wearable devices: a systematic literature review. Int. J. Med. Inform. 173, 105026. 10.1016/j.ijmedinf.2023.105026 36893657

